# Retraction: Anomaly detection in multivariate time series data using deep ensemble models

**DOI:** 10.1371/journal.pone.0326983

**Published:** 2025-07-01

**Authors:** The PLOS One Editors

After this article [1] was published, the following concerns were raised:

- The article is similar to a prior publication ([2]; cited as Reference 11 in [1]) that also evaluates methods for detecting anomalies in multi-dimensional time series data and that uses one of the same datasets as [1]. The related article was not declared at the time of submission as is required by PLOS policy.
- The Proposed Methodology and Experiments sections in [1] report content that was published in [2], the content reuse was not acknowledged in [1], and the prior publication [2] was not cited as the source.
- There are numerous instances in which the article uses terminology that differs from standards in the field. Examples include:
  - Center i7 instead of Core i7
  - Intel(R) Center instead of Intel(R) Core
  - LSTM organization instead of LSTM network
- There are concerns about the reliability and integrity of the article’s peer review.
- The article [1] cites an article ([3,4]; reference 18 in [1]) that was retracted prior to the publication of [1] due to concerns about manipulation of the publication process.

The corresponding author stated that departures from established terminology and phrasing were unintentional and the result of systematic errors and typographical oversights. They also stated that [1] and [2] are different in terms of methodology, results, and performance evaluation, noting that [1] is a direct continuation of [2], that [2] focuses on forecasting combined with anomaly identification by introducing an initial framework, and that [1] focuses on experimentation using ensembled deep learning methods and provides an advancement framework.

Members of the *PLOS One* Editorial Board assessed [1,2], and the authors’ responses. They confirmed there is overlapping content in [1] and [2], and noted that there are some differences in forecasting part and type and in the experimental results, and an additional dataset is used in [1].

The *PLOS One* Editors concluded that the article [1] does not comply with PLOS policies on Plagiarism (text reuse) and Submission and Publication of Related Studies. Furthermore, the article does not meet the journal’s reporting standards due to the extent of the issues with the language and terminology, which raise concerns about undisclosed AI use and may hinder readers’ understanding and interpretation of the content. In light of these issues and the peer review concerns, the *PLOS One* Editors retract this article [1].

PLOS regrets that these issues were not identified prior to publication.

AI and RA did not agree with the retraction. FSA and AA either did not respond directly or could not be reached.
